# Hsp90ab1 stabilizes LRP5 to promote epithelial–mesenchymal transition via activating of AKT and Wnt/β-catenin signaling pathways in gastric cancer progression

**DOI:** 10.1038/s41388-018-0532-5

**Published:** 2018-10-10

**Authors:** Huanan Wang, Guangxu Deng, Meiling Ai, Zhijun Xu, Tingyu Mou, Jiang Yu, Hao Liu, Shuang Wang, Guoxin Li

**Affiliations:** 1 Department of General Surgery, Nanfang Hospital, Southern Medical University, Guangdong Provincial Engineering Technology Research Center of Minimally Invasive Surgery, Guangzhou, 510515 China; 2 Department of Pathology, Southern Medical University, Nanfang Hospital, Guangzhou, 510515 China; 3 Department of Radiotherapy, Affiliated Cancer Hospital & Institute of Guangzhou Medical University, Guangzhou, 510515 China; 4 Department of Pathology, School of Basic Medical Sciences, Southern Medical University, Guangzhou, 510515 China

**Keywords:** Gastric cancer, Oncogenes

## Abstract

Hsp90ab1 is upregulated in numerous solid tumors, which is thought to induce the angiogenesis and promote cancer metastasis. However, it’s actions in gastric cancer (GC) has not been exhibited. In this study, Hsp90ab1 was demonstrated to be overexpressed and correlated with the poor prognosis, proliferation and invasion of GC. Ectopic expression of Hsp90ab1 promoted the proliferation and metastasis of GC cells both in vitro in cell line models of GC and in vivo using two different xenograft mouse models, while opposite effects were observed in Hsp90ab1 silenced cells. Moreover, the underlining molecular mechanism was explored by the co-immunoprecipitation, immunofluorescence, GST pull-down and in vitro ubiquitination assay. Namely, Hsp90ab1 exerted these functions via the interaction of LRP5 and inhibited ubiquitin-mediated degradation of LRP5, an indispensable coreceptor of the Wnt/β-catenin signaling pathway. In addition, the crosstalk between Hsp90ab1 and LRP5 contributed to the upregulation of multiple mesenchymal markers, which are also targets of Wnt/β-catenin. Collectively, this study uncovers the details of the Hsp90ab1-LRP5 axis, providing novel insights into the role and mechanism of invasion and metastasis in GC.

## Introduction

Gastric cancer (GC) is the fifth most common cancer globally and the third most common cause of cancer-based deaths in both males and females [1]. In China, GC ranks second among cancer deaths, and the incidence of GC is continually increasing [2–4]. Despite advances in both the diagnosis and therapy for GC in the past decade, GC survival has not markedly improved. Clinical evidence has demonstrated that the early development and dissemination of micro-metastatic cells may be responsible for tumor relapse and metastasis [5, 6]. However, little is known about the exact molecular mechanisms responsible for GC metastasis.

Accumulating evidence has shown that there is a close relation between the epithelial-mesenchymal transition (EMT) and cancer metastasis [7–9]. Multiple signaling pathways have been reported to be involved in EMT, including the AKT/mTOR, Wnt and AMPK pathways [10–14]. For example, prior researches have demonstrated that in non-small cell lung cancer and GC, tumor cells exhibit loss of the adhesion molecule biomarker E-cadherin and acquire the expression of mesenchymal biomarkers Vimentin or N-cadherin, proteins involved in the EMT, by the activation of TGF-β or Wnt/β-catenin [13, 15]. Therefore, we must explore key molecules involved in the invasion and metastasis, which may provide new insights into therapeutic targets.

The heat shock proteins are among the most abundantly expressed proteins in mammalian cells, and have previously been reported to have a role in the regulation of the tumorigenesis [16–20]. The heat shock proteins form a multiprotein chaperone complex which mediated the correct folding and stabilization of substrates involved in the cell cycle, proliferation, migration, and apoptosis [20–23]. In mammalian cells, there are four isoforms of Hsp90: Hsp90aa1, Hsp90ab1, GRP94, and TRAP1 [24], and Hsp90aa1 is the most well-studied one among them [25]. A recent study showed that Hsp90 inhibition prevented from proper folding and stabilization of its substrates, which resulted in the ubiquitination and degradation of the clients by the proteasome pathway [18].

Previous researches have demonstrated that Hsp90 promotes tumorigenesis in GC, breast cancer, non-small cell lung cancer, hepatocellular carcinoma, and conjunctival melanoma [24, 26–28]. Hsp90 overexpression has been associated with decreased survival in these cancer patients as well [29]. Hsp90 may promote tumorigenesis in part due to its increased affinity for ATP and ATPase activity in cancer cells [16–20]. It is also reported that Hsp90ab1 overexpression promotes the angiogenesis, metastasis and differentiation of hepatocellular carcinomas and lung cancer [24, 30]. In other tumors, numerous studies have demonstrated that Hsp90aa1 participates in tumorigenesis. However, the role of Hsp90ab1 in GC carcinogenesis has not been elucidated comprehensively so far.

Here, we hypothesized that Hsp90ab1 promotes GC metastasis, leading to a worse prognosis, by activation of the EMT. We began by investigating the relationship of Hsp90ab1 with patient survival. Subsequently, in order to better understand the mechanisms of underlying GC progression, we used GC cell lines and mouse models to elucidate the role of aberrant Hsp90ab1 expression in GC tumorigenesis. Finally, we confirmed that Hsp90ab1 stabilized LRP5 to promote EMT via activating the AKT and Wnt/β-catenin signaling pathways.

## Results

### Up-regulation of Hsp90ab1 in GC tissues correlates with GC metastasis

**Fig. 1:**
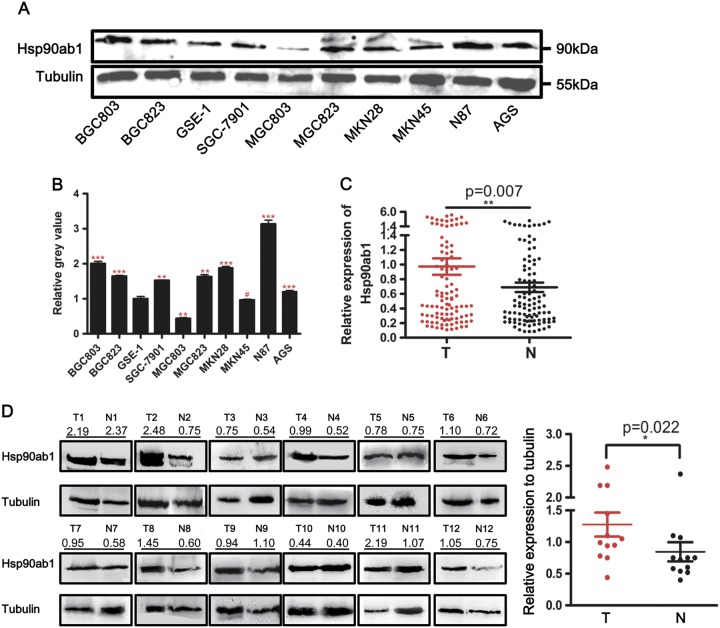
Hsp90ab1 expression was up-regulated in GC cell lines and patients. **a** Expression levels of Hsp90ab1 were determined by Western blotting in GC and gastric mucosa epithelial cell lines. **b** The relative protein expression levels were quantified by using Quantity One Software, and the relative protein abundance of Hsp90ab1 in the individual cells was calculated by normalization with Tubulin expression. **c** Expression of Hsp90ab1 in 102 paired human GC tissues by q-PCR, which was determined by normalization with GAPDH control. **d** Expression analyses of Hsp90ab1 protein in the 12 paired gastric carcinomas samples by Western blotting. *N* normal mucosa, *T* tumor. The protein expression levels were quantified by Quantity One Software, and the relative protein abundance was determined by normalization with Tubulin. Error bars represented the mean ± SD of three replicates. #*p* > 0.05; *p < 0.05; **p < 0.01; ***p < 0.001

**Fig. 2:**
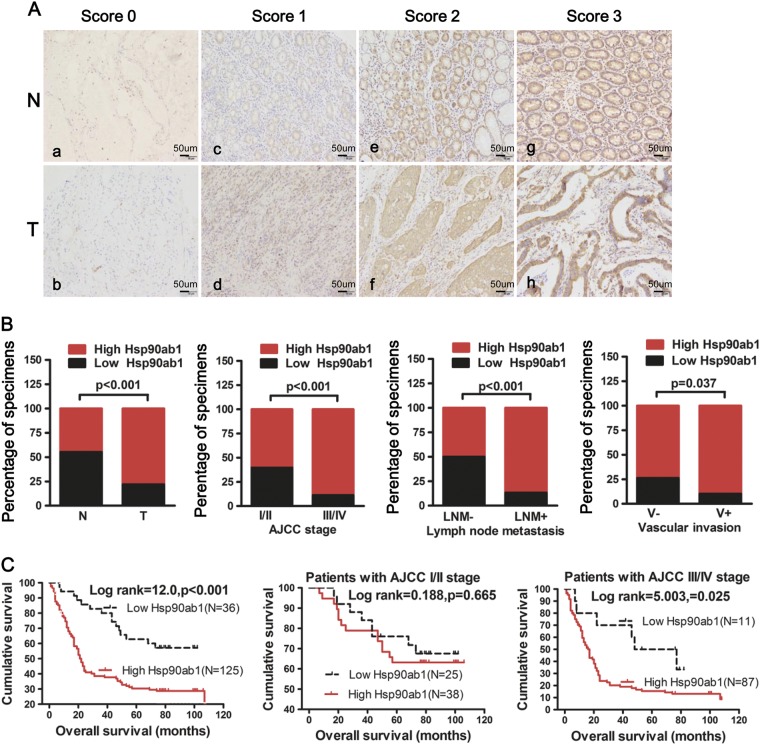
Hsp90ab1 expression was positively associated with progression and poor prognosis and could be an independent prognostic factor for GC patients. **a** IHC analysis of Hsp90ab1 expression in GC tissue samples (T) and surrounding non-tumor tissues (N). The representative figures of GC tissues with low and high Hsp90ab1 levels of the staining intensity were shown. **b** Graphical illustration of statistical Hsp90ab1 distribution in GC tissues. Hsp90ab1 was significantly higher in GC than their adjacent nontumorous tissues. Hsp90ab1 protein expression was more frequently found in GC with AJCC classification (III/IV) than that with AJCC classification (I/II). Also Hsp90ab1 protein expression was more frequently found in GC with lymph node metastasis, vascular invasion than that in GC without lymph node metastasis, vascular invasion. **c** Kaplan–Meier survival analysis for GC patients with distinct expression level of Hsp90ab1 and AJCC classification after surgical resection

We originally compared the expression of Hsp90ab1 mRNA and protein in a panel of GC cell lines to gastric mucosa epithelial cell line (GSE-1). Seven out of nine GC cells had increased Hsp90ab1 protein expression (Fig. 1a, b) to varying degrees. Additionally, six GC cell lines showed higher mRNA expression level of Hsp90ab1 compared to GSE-1 cells (Figure S1A). To confirm whether Hsp90ab1 was also upregulated in GC, Hsp90ab1 mRNA and protein expression was assessed in patient samples of GC tissues and adjacent normal gastric mucosa tissues removed during radical resection. The level of Hsp90ab1 mRNA was dramatically upregulated in the 102 GC tissues compared with their paired normal samples (Fig. 1c, Figure S1B, *P* < 0.01). In addition, in the 12 sample pairs, Hsp90ab1 protein expression was significantly higher in the GC tissues than that in the normal gastric samples (Fig. 1d, *P* *<* 0.05). Finally, we performed IHC analysis of a tissue microarray. Hsp90ab1 expression was up-regulated in 135 cases and downregulated in the other 38 cases in the GC tissue samples, whereas the positive rate of Hsp90ab1 was significantly low in the normal gastric mucosa (Fig. 2a, b, *χ*^2^ = 49.237, *P* < 0.001). Taken together, these results indicated that Hsp90ab1 was up-regulated in GC.

To explore whether Hsp90ab1 expression level was related to GC progression, we analyzed the association between Hsp90ab1 and clinicopathologic status in 161 GC patients with complete pathological and follow-up data. Hsp90ab1 protein expression level was positively associated with lymph node metastasis (Fig. 2b, *χ*^2^ = 23.421, *P* *<* 0.001), vascular invasion (Fig. 2b, *χ*^2^ = 4.343, *P* *<* 0.05), American Joint Committee on Cancer (AJCC) stage (Fig. 2b, *χ*^2^ = 17.889, *P* *<* 0.001) and T stage (Table S3, *χ*^2^ = 6.313, *P* < 0.05). However, no significant correlation was found between Hsp90ab1 protein expression and other clinicopathological parameters (Table S3). Furthermore, overall survival of 161 patients with follow-up data displayed that higher Hsp90ab1 expression was significantly correlated with reduced overall survival in GC patients (Fig. 2c, log-rank test = 12.0, *P* *<* 0.001), which was supported by public data from Kaplan–Meier plotter using Gene Expression Omnibus and TCGA datasets (Figure S1C-D) (KM plotter, http://kmplot.com)[31, 32]. Furthermore, multivariate survival analysis suggested that Hsp90ab1 expression [*P* *=* 0.017; HR = 1.983; 95% confidence interval (CI), 1.130–3.478], AJCC stage (*P* *<* 0.001; HR = 4.166; 95% CI, 2.573–6.746) and vascular invasion (*P* *=* 0.001; HR = 1.524; 95% CI, 1.177–1.974) were confirmed to be independent prognosis factors of disease outcome in GC patients (Table 1). Overall, these findings strongly suggested that Hsp90ab1 expression was associated with GC invasion and migration.

**Table1:** Univariate and multivariate analysis of different prognostic parameters for GC patients

Variables	HR(95%CI)	*P* value
*Univariate analysis*		
Gender (M vs F)^a^	1.440(0.975–2.128)	0.067
Age(years)(<60 vs ≥60)	0. 955(0.643–1.418)	0.818
Location (C/B vs A/W)^b^	0.826(0.556–1.228)	0.345
Tumor size (cm in diameter)(>5.5 vs ≤5.5)	1.707(1.127–1.945)	**0.007**
Vascular invasion (yes vs no)	1.481(0.671–2.155)	**0.005**
Histological stage (P/U vs W/M)^c^	2.121(1.259–3.573)	**0.005**
AJCC stage (I/II vs III/IV)	4.559(2.845–7.306)	**<0.001**
T stage (T1/T2 vs T3/T4)	3.632(1.761–7.492)	**<0.001**
N stage (N0 vs N1~ 3)	3.992(2.224–7.166)	**<0.001**
M stage (M0 vs M1)	2.613(1.379–4.952)	**0.003**
Hsp90ab1 expression (low vs high)	2.525(1.457–4.377)	**0.001**
*Multivariate analysis*		
AJCC stage (I/II vs III/IV)	4.166(2.573–6.746)	**<0.001**
Vascular invasion (yes vs no)	1.524(1.177–1.974)	**0.001**
Hsp90ab1 expression (low vs high)	1.983(1.130–3.478)	**0.017**

*HR* hazard ratio, *CI* confidence interval

*Statistically significant (*P* < 0.05)

^a^*M* male, *F* female

^b^*C* cardia, *B* body, *A* antrum, *W* whole

^c^*P* poor, *U* undifferentiated, *W* well, *M* moderate

### Knockdown of Hsp90ab1 represses GC cell proliferation, invasion, and migration in vitro

**Fig. 3:**
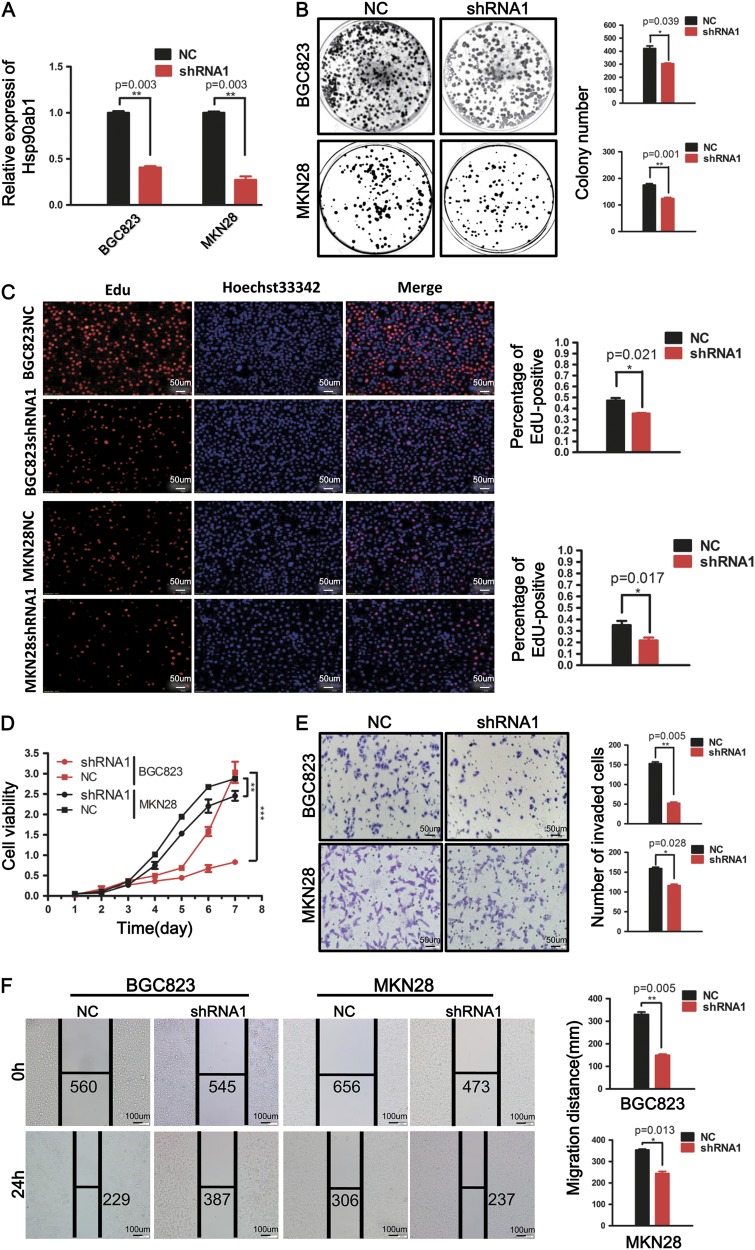
Silencing of Hsp90ab1 inhibited cell proliferation, invasion, and migration in vitro. **a** Hsp90ab1 mRNA in BGC823 and MKN28 cells after shRNA-mediated knockdown of Hsp90ab1 was detected by real-time RT-PCR. **b**–**d** Down-regulation of Hsp90ab1 resulted in inhibiting the proliferation and DNA replication of BGC823 and MKN28, as detected by colony formation assays (**b**), EdU incorporation assay (**c**) and CCK-8 assays (**d**). **e** The invasion ability of GC cells was significantly reduced in Hsp90ab1-depleted cells compared with the control groups, as revealed by Matrigel invasion assays. **f** Down-regulation of Hsp90ab1 reduced the migration ability of GC cells, as determined by Scratch-wound-healing assays. The data was expressed as the mean ± SD and reproduced in three independent experiments. *P < 0.05; **P < 0.01; ***P < 0.001

To verify if Hsp90ab1 is necessary for GC oncogenesis, endogenous Hsp90ab1 expression was silenced in BGC823 and MKN28 cells. qRT-PCR analysis proved a significant decrease of Hsp90ab1 expression in the shRNA1 group compared with the control group (Fig. 3a, *P* *<* 0.01). The capacity to form colonies in the Hsp90ab1-depleted cells was remarkably suppressed compared with the cells transfected with control vectors (Fig. 3b, *P* *<* 0.05). Similarly, we measured cell proliferation visualizing the incorporation of EdU into DNA. Depletion of Hsp90ab1 significantly reduced cell proliferation as compared to controls (Fig. 3c, *P* *<* 0.05). Finally, the CCK-8 assay revealed that the downregulation of Hsp90ab1 could markedly inhibit GC cell growth compared with the negative control (Fig. 3d, *P* *<* 0.01). We also performed colony formation assays, CCK-8 cell proliferation assays and EdU staining with shRNA2 in BGC823 and MKN28 cell lines, respectively. Our data were consistent with our previous results (Figs. S2B-2D, *P* < 0.05). Finally, the knockdown of Hsp90ab1 in GC cells significantly reduced both cell invasion and migration capacity, as illustrated by Matrigel invasion assay and wound healing assay (Fig. 3e, f, Figure S2E, *P* < 0.05). These data strongly suggested that Hsp90ab1 was essential for the invasion and migration of GC cells.

Besides, to validate whether the Hsp90ab1-depletion suppresses progression through cell cycle, cellular DNA content were quantified by flow cytometry. The depletion of Hsp90ab1 caused a significant reduction of DNA content, suggesting impeded S phase progression (Figure S3A). These results indicated that the downregulation of Hsp90ab1 inhibited the proliferation of GC cells in vitro.

### Ectopic overexpression of Hsp90ab1 promotes GC cell proliferation, invasion and migration and tolerance to chemotherapeutic drugs in vitro

**Fig. 4:**
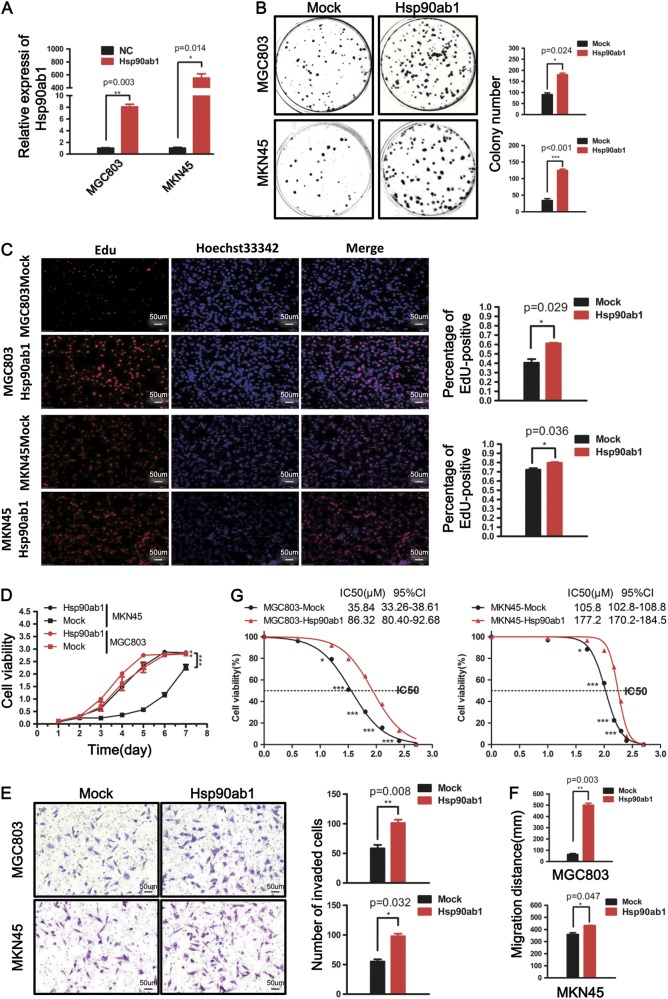
Overexpression of Hsp90ab1 promoted cell proliferation, invasion and migration and tolerance to chemotherapeutic drugs in vitro. **a** Expression analyses of Hsp90ab1 mRNA in MGC803 and MKN45 cells after lentivirus-mediated overexpression of Hsp90ab1. **b**–**d** Elevated expression of Hsp90ab1 induced significantly higher growth rates, DNA replication and proliferation of MGC803 and MKN45 in GC cells. **e** The invasion ablity of GC cells was enhanced in Hsp90ab1-overexpressing cells compared with the control groups. **f** Up-regulation of Hsp90ab1 promoted the migration ability of GC cells. **g** Growth inhibitory effect of oxaliplatin on Hsp90ab1-overexpressing and empty vector-transfected MGC803 and MKN45 cells. The data was expressed as the mean ± SD and reproduced in three independent experiments. *P < 0.05; **P < 0.01; ***P < 0.001

To show Hsp90ab1 is sufficient to promote cell proliferation and migration, we adopted gain-of-function models with MKN45 and MGC803 cells. To confirm the overexpression of Hsp90ab1, we measured mRNA levels after transfection with Hsp90ab1 lentiviral vectors. Transfected cells significantly upregulated Hsp90ab1 as compared to control cells (Fig. 4a, *P* *<* 0.01). As revealed by colony formation assays, GC cells that overexpressed Hsp90ab1 had a dramatically increased growth rate as compared to mock-transfected cells, indicated by increased formation of colonies (Fig. 4b, *P* *<* 0.05). Additionally, Hsp90ab1 overexpression in both cell lines resulted in increased cell proliferation as measured by an EdU incorporation assay (Fig. 4c, *P* *<* 0.05). Similarly, the increased cell numbers were also observed in the Hsp90ab1 overexpression group, indicated by CCK-8 (Fig. 4d, *P* *<* 0.01). Finally, the proportion of cells in S phase was increased and the proportion of cells in G1 phase was decreased to a comparable degree (Figure S3B). These results indicated Hsp90ab1 promoted cell growth by promoting cell cycle progression.

To determine the effects of Hsp90ab1 on cell migration and invasion, we observed the effect of Hsp90ab1 overexpression by a Matrigel invasion assays and a wound closure assay. Significantly higher migration rates and more rapid wound closures were observed in the group of Hsp90ab1-overexpressing compared with the control (Fig. 4e, f, Figure S3C). Collectively, these data demonstrated that Hsp90ab1 expression induced GC cell proliferation, invasion, and migration.

To investigate the effects of Hsp90ab1 expression on the chemosensitivity of gastric cancer cells to chemotherapeutic agents, MGC803 and MKN45 cells stably transfected with Hsp90ab1 lentiviral vectors and empty vector were treated with oxaliplatin at different concentrations for 24 h. As revealed in Fig. 4g, Hsp90ab1 significantly increased the tolerance of MGC803 and MKN45 cells to oxaliplatin.

### Hsp90ab1 promotes the growth and metastasis of human GC cell in vivo

**Fig. 5:**
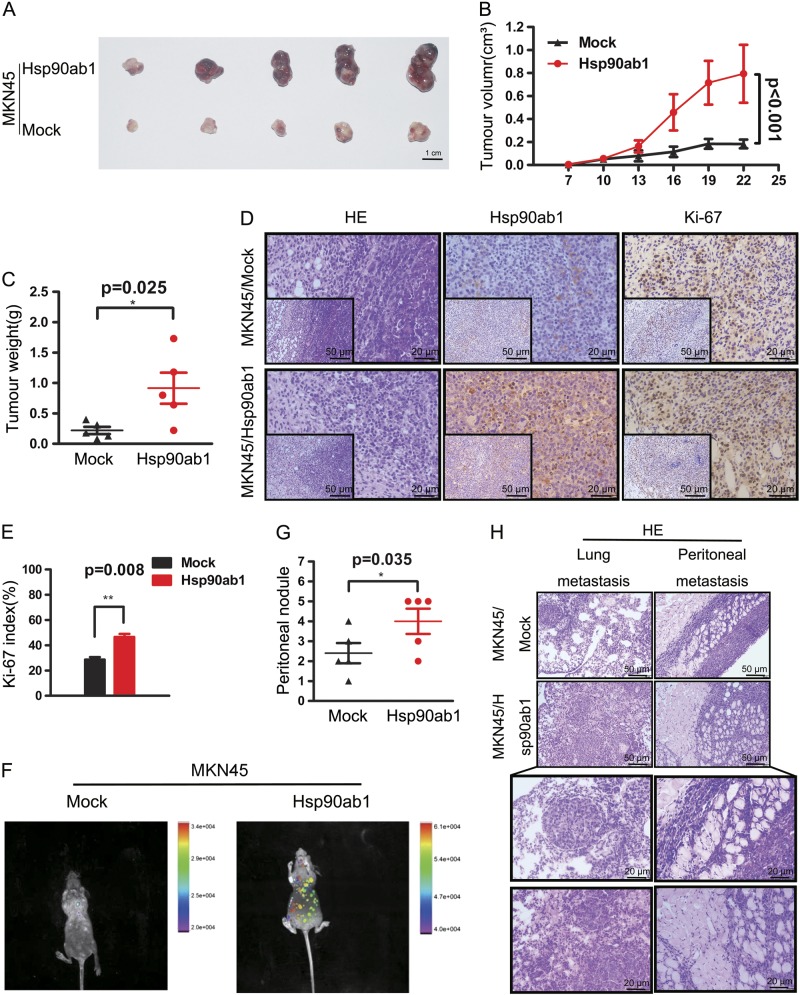
The Hsp90ab1 overexpression promoted the growth and metastasis of GC in vivo. **a**–**c** Images of subcutaneous tumors of mice injected with Hsp90ab1-overexpression and control stable transfected cells. **a** MKN45 cells with upregulated Hsp90ab1 expression exhibited enhanced tumor growth in nude mice. **b** The effect of Hsp90ab1 on GC tumor growth was evaluated based on tumor volume injected with indicated cells. The data of subcutaneous tumors were expressed as mean ± SD. **c** Scatter plots of tumor weight derived from indicated cells after subcutaneous implantation. **d**, **e** The xenograft tumors was stained with H&E and the expression of Hsp90ab1 and Ki-67 in the tumors was measured by IHC. Proliferative ability was measured by the Ki-67 index (%). **f**–**h** Hsp90ab1 upregulated cells or control cells were injected into nude mice through the tail vein to evaluate the metastatic potential of cells. The infection efficacy of lentivirus with GFP in vivo (**f**) and number of metastatic peritoneal nodules (**g**) in individual mice was analyzed. **h** The lung metastasis and metastatic peritoneal nodules sections were stained with (H&E). Bottom pictures were local magnification of top pictures. *P < 0.05; **P < 0.01; ***P < 0.001

Then we sought to demonstrate that Hsp90ab1 promotes GC development in vivo. Therefore, we generated xenograft models of GC by subcutaneously injecting GC cells stably transfected with Hsp90ab1 and empty vector into nude mice. Mice with Hsp90ab1-overexpressing cells developed significantly larger tumors than those transfected with control cells (Fig. 5a, b). The tumors that developed from Hsp90ab1-transfected GC cells weighed significantly more than the controls at the termination of the experiment (Fig. 5c, *P* *<* 0.05). Hematoxylin and eosin staining (H&E) revealed that the Hsp90ab1-transfected tumors had similar histopathological features to human GC samples (Fig. 5d). To quantify cell growth in the tumor samples, we visualized Hsp90ab1 and Ki-67 expression in paraffin-embedded xenograft tumors. In concordance with the findings in vitro, significantly higher expression of Ki-67 was detected in Hsp90ab1 overexpressing tumors than that in the control tumors (Fig. 5d, e, *P* *<* 0.01).

To determine if Hsp90ab1 play a role in GC metastasis in vivo, we next generated metastatic mouse models. MKN45 cells with and without stable overexpression of Hsp90ab1 were injected into the tail vein of nude mice. The infection efficacy of the lentivirus in vivo was confirmed by imaging mice on the animal in vivo imaging instrument (Fig. 5f). Mice injected with Hsp90ab1 expressing cells developed more lung metastases and peritoneal metastases compared to those with the control cells (Fig. 5g, h). These results corroborated previous data and suggested that Hsp90ab1 promoted tumor growth and metastasis in GC cells.

### Hsp90ab1 directly interacts with LRP5 and positively regulates LRP5 expression in GC cells

**Fig. 6:**
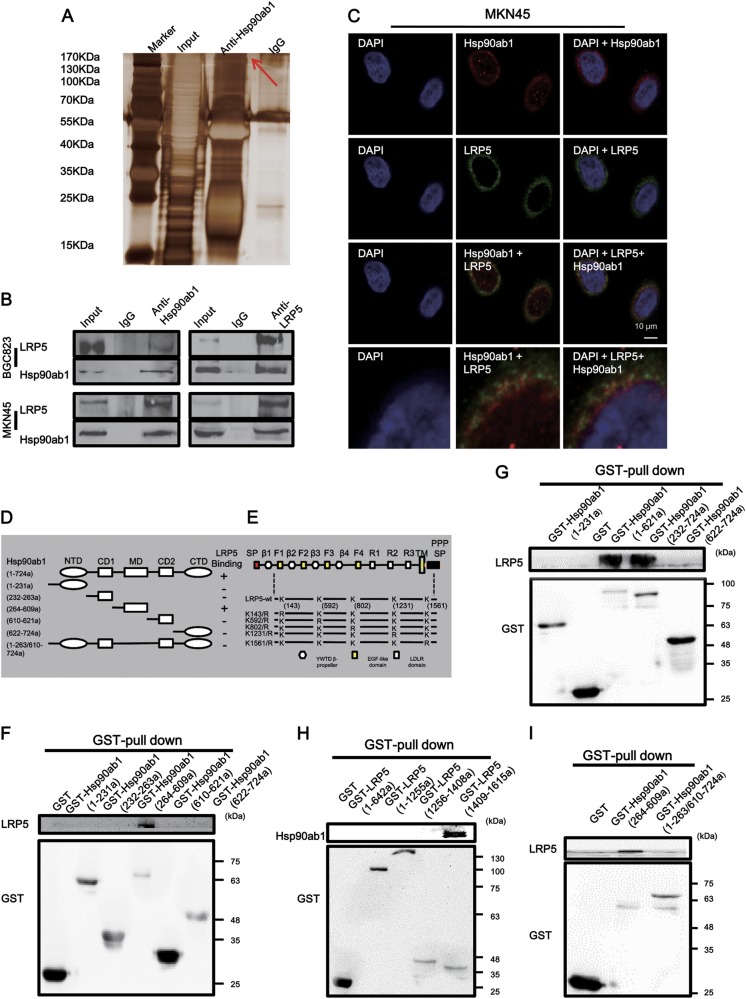
Hsp90ab1 interacted with LRP5 protein in GC. **a** Immunoprecipitation assays were used to identify proteins associated with Hsp90ab1. The anti-Hsp90ab1 and IgG antibody were incubated with cell extracts, the bands specific were excised and submitted for mass spectrometry, and LRP5 was identified to be a Hsp90ab1-binding protein. **b** Co-IP of Hsp90ab1 and LRP5 with each other from proteins of BGC823 and MKN45 cells in GC cells. **c** Confocal immunofluorescence analysis showed the presence and localization of Hsp90ab1 and LRP5 in the cytoplasm of MKN45 cells. **d**, **e** Schematics outlines of both Hsp90ab1 and LRP5 structure features. **f**–**h** GST pull-down assay results indicated the direct interaction between Hsp90ab1 and LRP5. **i** Pull-down of LRP5 by GST-tagged Hsp90ab1 fragment (264–609a) and Hsp90ab1 fragment (1–263/610–724a)

In order to gain a better understand of the molecular mechanism of Hsp90ab1 in GC cells, we first sought to identify the proteins that directly interacted with Hsp90ab1 in BGC823 cells by immunoprecipitation (IP). The most abundant protein isolated was low-density lipoprotein receptor-related protein 5 (LRP5), as identified by mass spectrometry (Fig. 6a, Table S4). To confirm the protein–protein interaction between Hsp90ab1 and LRP5, we conducted a Co-IP analysis with an antibody against Hsp90ab1. After immunoprecipitation with Hsp90ab1 conjugated beads, LRP5 was detected in the lysates from both BGC823 and MKN45 cells. In a reciprocal Co-IP with LRP5 conjugated beads, Hsp90ab1 precipitated with LRP5, indicating that endogenous human Hsp90ab1 was physically associated with LRP5 (Fig. 6b). Immunofluorescent staining revealed that Hsp90ab1 and LRP5 co-localized in both cell lines at the cell membrane and in the cytosol (Fig. 6c, Figure S4). Schematic of Hsp90ab1 and LRP5 show the domain of Hsp90ab1 and LRP5 (Fig. 6d, e). Then we performed GST pulldown assays according to this schematic and found that the MD domain of Hsp90ab1 interacted with the domain (1409–1615a) of LRP5 (Figs. 6f-h). Finally, we also generated MD domain deletion constructs and found that the deletion of MD fragment within Hsp90ab1 abolished the ability of Hsp90ab1 to interact with LRP5 by GST pulldown assays and exhibited a weak effect on promoting tumor aggressiveness compared with the overexpression group in vitro experiments (Fig. 6i, Figure S5).

**Fig. 7:**
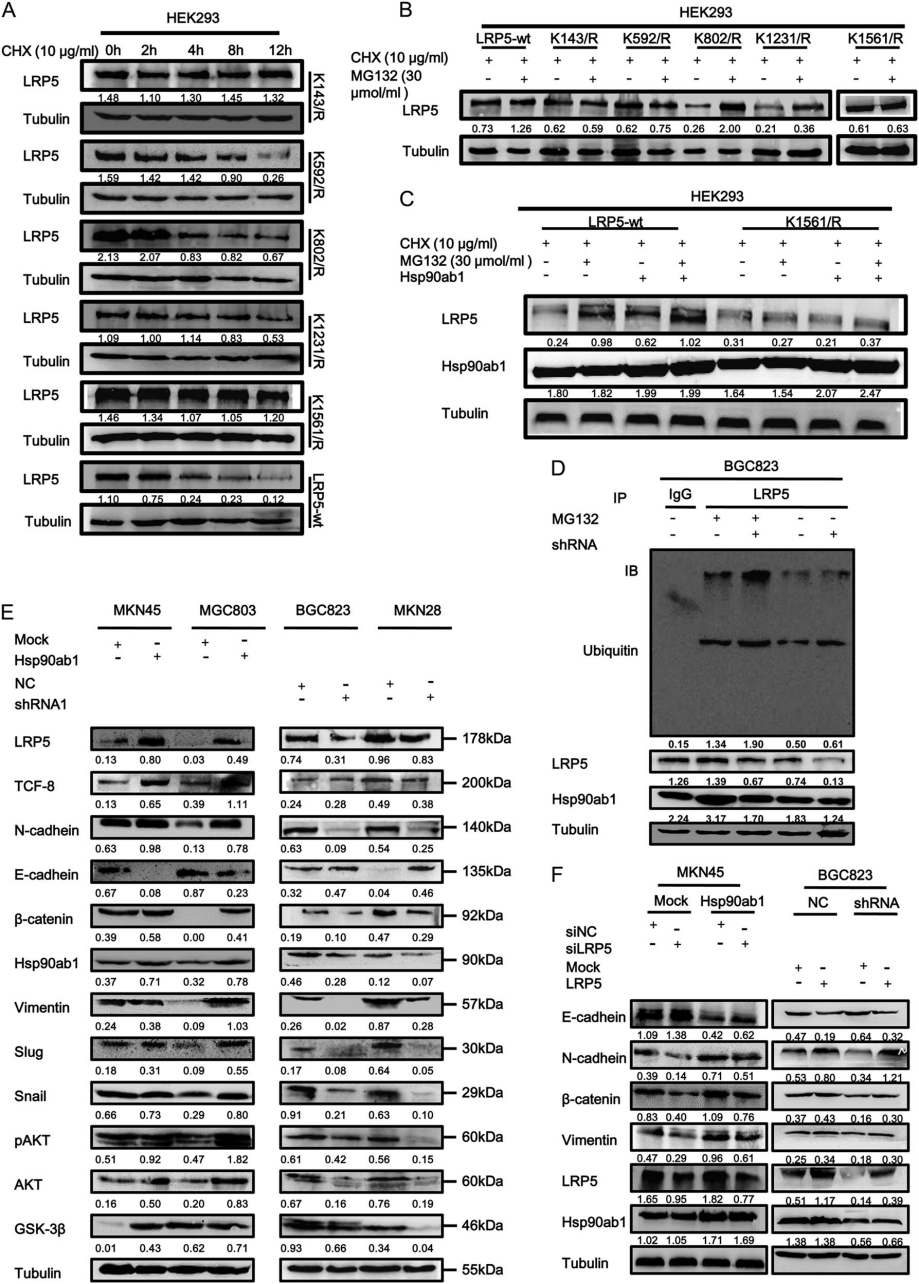
Hsp90ab1 inhibited the ubiquitin degradation of LRP5 and promoted the proliferation and invasion of GC cells by activating AKT and Wnt/β-catenin signaling pathway. **a** HEK 293 T cells were transiently transfected with LRP5-wt plasmid or the K142R, K592R, K802R, K1231R, and K1561R mutants, and treated with CHX at 10 μg/ml for the indicated times. Cell lysates were assessed by Western blot analysis. **b** HEK 293 T cells were transiently transfected with LRP5-wt plasmid or the K142R, K592R, K802R, K1231R and K1561R mutants, and treated with CHX at 10 μg/ml and MG132 at 30 μM for 12 h. Cell lysates were assessed by Western blot analysis. **c** HEK 293 T cells were transiently co-transfected with LRP5-wt plasmid or the K1561R mutants and Hsp90ab1, and treated with CHX at 10 μg/ml and MG132 at 30 μM for 12 h. Cell lysates were assessed by Western blot analysis. **d** Hsp90ab1 increased the LRP5 level by inhibiting its ubiquitin-mediated degradation in BGC823 cells. **e** Western blotting analyses of the levels of Akt, Wnt/β-catenin signaling pathways and classical EMT markers in GC cells treated with Hsp90ab1 or shRNA1. **f** Western blotting analyses of the levels of Hsp90ab1, LRP5 and classical EMT markers in GC cells treated with co-transfection of Hsp90ab1 and siLRP5 or shHsp90ab1 and LRP5

When we analyzed the protein sequence of LRP5 in the Compendium of Protein Lysine Modifications database, some ubiquitination sites were predicted within LRP5 (Figure S6). To determine if the protein is ubiquitinated and degraded, we inhibited the synthesis of LRP5 and site-mutated proteins with cycloheximide in HE293 cells. Overexpression of the K143R, K592R and K1561R mutants were more stable than the K802R and K1231R mutants, compared with LRP5-wt, indicating that the Lys-to-Arg mutations at these sites abolish LRP5 degradation more or less (Fig. 7a). Then we examined whether MG132, a proteasome inhibitor, could reverse the effect, results of western blot showed that the protein content of LRP5-wt, K802R and K1231R increase obviously (Fig. 7b). Therefore, we believed that K143R, K592R and K1561R were potential Ub acceptor sites which mediated LRP5 ubiquitination by the proteasomal pathway. Because Hsp90ab1 and LRP5 interacted with each other in the domain (1409–1615a) of LRP5, we focused on the effect of K1561R site mutation on LRP5. Results of western blot showed that both MG132 and Hsp90ab1 did not increase the LRP5 protein level in HEK293 cells compared with the control group (Fig. 7c).

In order to better understand the relationship between Hsp90ab1 and LRP5 in GC cells, we measured LRP5 protein levels in cell lines after lentiviral modulation of Hsp90ab1 expression. In BGC823 cell, transfected with shRNA to knock down Hsp90ab1, LRP5 protein levels decreased as compared to controls (Fig. 7d). We hypothesized that the interaction of Hsp90ab1 and LRP5 sequesters LRP5, which prevents its ubiquitination and proteasome-mediated degradation. To confirm that LRP5 expression is affected by ubiquitination pathway mediated by Hsp90ab1, we utilized an in vitro ubiquitination assay to visualize the level of LRP5 ubiquitination. Lentivirus-mediated downregulation of Hsp90ab1 resulted in decrease in LRP5 protein levels. To confirm this is due to protection from inhibiting the ubiquitin mediated degradation, cells treated with 30 μM MG132, resulted in accumulation of LRP5 (Fig. 7d). These above data suggested that stabilization of LRP5 was partly mediated by the Hsp90ab1-LRP5 interaction, which inhibited the ubiquitin-mediated degradation of LRP5.

### Hsp90ab1-mediated EMT in GC cell is mediated by LRP5-induced activation of the AKT and Wnt/β-catenin signaling pathways

We next sought to understand how Hsp90ab1-mediated protection of LRP5 affected GC cell behavior. LRP5 is a known activator of Wnt/β-catenin signaling pathway, which promotes cell growth and migration [33]. Therefore, we hypothesized that overexpression of Hsp90ab1 would increase the levels of β-catenin and phosphorylated AKT, while knocking down Hsp90ab1 would decrease β-catenin and phosphorylated AKT. After Hsp90ab1 overexpression, both the levels of phosphorylated AKT and β-catenin were obviously increased, indicating increased pathway activation. Alternately, knockdown of Hsp90ab1 showed the opposite results. It has been widely known that Wnt/β-catenin activation induces the EMT. Therefore, we hypothesized that the expression of Hsp90ab1 would also change the levels of classical EMT markers, such as E-cadherin, N-cadherin, Vimentin, TCF8, Snail, and Slug. We measured levels of these proteins by western blotting in MKN45 and MGC803 cells with up-regulated Hsp90ab1 expression. These cells had increased abundance of N-cadherin, Vimentin, TCF8, Snail, and Slug, but reduced protein abundance of E-cadherin. Hsp90ab1-depleted cells displayed the opposite results (Fig. 7e, Figure S7A). Similarly, knockdown of Hsp90ab1 showed the reduction of LRP5 and β-catenin, as revealed by immunofluorescence, while overexpression of Hsp90ab1 reverse the effect (Figure S7A, B, Table S5).

**Fig. 8:**
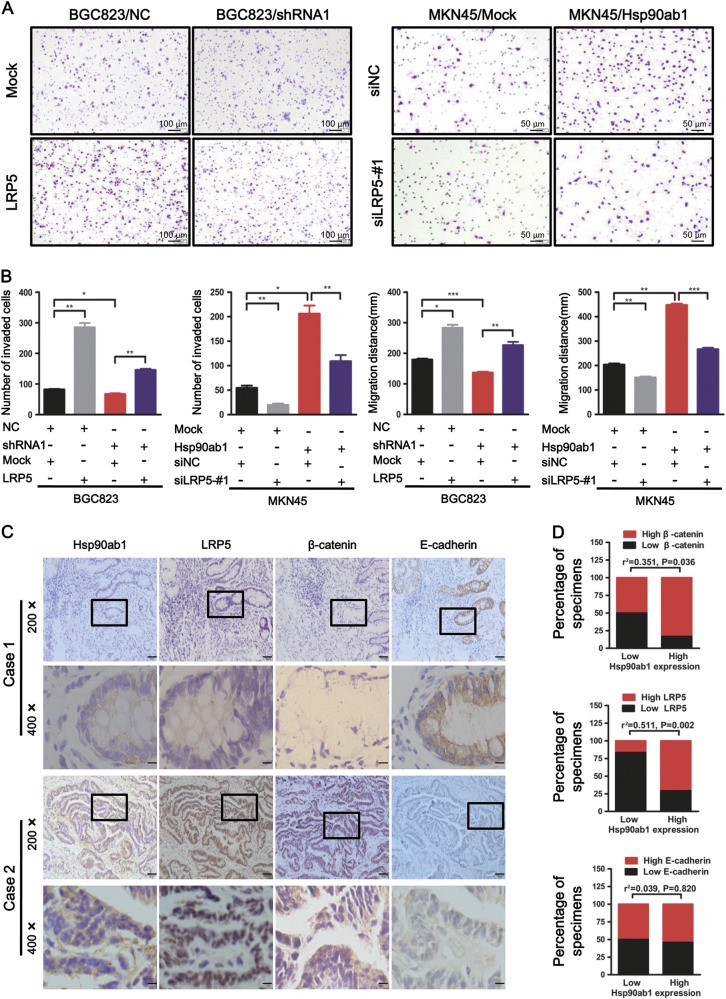
LRP5 was positively correlated with Hsp90ab1 in GC cells and tissues. **a** Effects of co-transfection of shRNA1 and LRP5 or Hsp90ab1 and siLRP5-#1 on cell invasion and migration by Matrigel invasion assays and Scratch-wound-healing assays. **b** Graphical illustration of statistical result of co-transfection of shRNA2 and LRP5 or Hsp90ab1 and siLRP5-#1 on cell invasion and migration. **c** The Hsp90ab1 expression was positively correlated with LRP5 and β-catenin, but it was meaningless correlation with E-cadherin in 36 GC specimens. Representative figures of two cases were shown. Bottom pictures were local magnification of top pictures. **d** Graphical illustration of the levels of Hsp90ab1 expression that were associated with the levels of β-catenin, LRP5, and E-cadherin in 36 GC specimens

To confirm the role of the Hsp90ab1-LRP5 axis in Hsp90ab1-mediated EMT, cell invasion, and metastasis, we hypothesized that LRP5 overexpression and knockdown would reverse the effects of Hsp90ab1 knockdown and overexpression in GC cells. When we induced LRP5 knockdown in Hsp90ab1 overexpressing cells, western blotting indicated reversed EMT markers occurred in these cells (Fig. 7f). To confirm this had a functional consequence, we also measured the migratory capacity of these cells. In alignment with EMT marker reversal, cell migration was also decreased. Alternately, LRP5 overexpression in Hsp90ab1-depleted cells reversed downregulation of EMT markers and impaired migratory activity of GC cells (Fig. 8a, b, Figure S8A, D).

Finally, we evaluated the expression of Hsp90ab1, LRP5, β-catenin and E-cadherin in tissue samples from GC patients. The expressions of Hsp90ab1, LRP5, β-catenin and E-cadherin were detected in 36 patients by immunohistochemistry (Fig. 8c, d). Hsp90ab1 levels in these samples were positively correlated with the levels of LRP5 and β-catenin (LRP5: *r*^2^ = 0.551, *P* = 0.002 and β-catenin: *r*^2^ = 0.351, *P* = 0.036). However, there was no significant correlation between Hsp90ab1 expression and E-cadherin (r^2^ = 0.039, *P* = 0.820). Taken together, these data supported the hypotheses that Hsp90ab1 promoted the invasion and migration of GC cells via the formation and activation of Hsp90ab1-LRP5 axis.

## Discussion

In the present study, we demonstrated that Hsp90ab1 was upregulated in GC cells, which resulted in significantly worse patient outcomes and higher cancer metastasis rate. We showed that this was a result of Hsp90ab1 mediated stabilization of LRP5, which activated both AKT and Wnt/β-catenin signaling pathways. It is well known that Hsp90ab1 is a molecular chaperone that is required for the formation, stability and function of its client proteins, many of which promote cancer cell growth and survival [23]. Growing evidence has revealed that Hsp90ab1 overexpression occurs in various different cancers, and this overexpression plays an integral role in the multistep processes leading to carcinogenesis and invasion [24, 30, 34, 35]. However, the significance and underlying mechanism of Hsp90ab1 in GC was previously unknown.

We first confirmed that Hsp90ab1 was remarkably up-regulated in GC tissues and it was significantly associated with aggressive stages and unfavorable prognosis of GC patients. By Kaplan–Meier survival curve analysis, we found that the disease-free survival of patients with Hsp90ab1 high expression was significantly shorter than that of other patients. In particular, the expression of Hsp90ab1 was significantly associated with shorter survival for patients with stage III/IV GC. Furthermore, we found that Hsp90ab1 was an independent prognostic factor for overall survival of GC patients, indicated by multivariate cox regression analysis. Besides, Hsp90ab1 could increase the tolerance of GC cells to chemotherapeutic agents, such as HIF1α, Cdc37, to affect the prognosis of patients[36, 37]. These data suggested that Hsp90ab1 might be a valuable new prognostic marker for the screening, diagnosis, and prognosis for GC patients.

Previously, Hsp90ab1 has been implicated to have an oncogenic role in laryngeal carcinoma [38], non-small-cell lung cancer [30, 39], and breast cancer [40, 41] by promoting tumorigenesis. In this study, we demonstrated that Hsp90ab1 was both necessary and sufficient to promote the invasion and migration of GC tumorigenesis in vitro and in vivo, thereby indicating an invasion-promoting effect in GC progression. In combination with prior findings, our data supported the hypothesis that Hsp90ab1 plays an important role in tumorigenesis in multiple tumor types.

Recently, it was proposed that Hsp90ab1 might stabilize Cdc25A, increasing its expression levels and promoting cell-cycle activation in pancreatic carcinoma cells [42]. Likewise, Hsp90ab1 has also been shown to promote leukemia cell proliferation by preventing the auto-ubiquitination and degradation of c-IAP1, a member of the inhibitor of apoptosis protein (IAP) family [43]. Beside, Hsp90 has been reported to interact with AKT to induce autophagy through activation of the AKT/mTOR pathway in lung cancer [44]. However, we suspect that apoptosis inhibition is not the only function of Hsp90ab1, and that its role in GC may be mediated by its role as a molecular chaperone.

In our study, we showed that LRP5 was a novel binding partner of Hsp90ab1 in GC cells. LRP5 has been reported to be an indispensable coreceptor that regulates the activity and function of Wnt/β-catenin signaling pathway [33]. LRP5 binds to Frizzled (Fz) and Dishevelled Segment Polarity Protein 2 (Dvl-2v) for the initiation of Wnt signaling [45, 46], which results in the translocation of the transcription co-activator protein, β-catenin, into the nucleus. Additionally LRP5 overexpression has previously been shown to increase β-catenin expression and activation in malignant cancers [47–49]. In the present study, we revealed that Hsp90ab1 reduced the ubiquitin-mediated proteasome degradation of LRP5, resulting in LRP5 upregulation in GC cells. We confirmed the role of LRP5 in GC by showing that knockdown of LRP5 partly counteracted the malignant phenotypes mediated by Hsp90ab1. However, the exact mechanisms dictating LRP5 ubiquitination and activation within GC cells require further exploration. Although further studies need to be carried out, these results collectively demonstrated that the role of LRP5 is essential in Hsp90ab1-mediated cancer metastasis.

The EMT occurs via activation of both AKT and and Wnt/β-catenin signaling pathways, which may contribute to tumor metastasis [49–52]. Our results illustrated that Hsp90ab1 was a new interacting partner of LRP5, an indispensable coreceptor of the Wnt signaling pathway. Enhanced canonical Wnt signaling increases the stabilization of β-catenin and induces the nuclear import of β-catenin, which increases the levels of transcription factors TCF, Snail, and Slug. These proteins regulate the activation of the EMT. Additionally, the EMT has previously been shown to be a positive regulator of growth, migration, and invasion in GC [53–55]. As confirmed by the current research, Hsp90ab1 can increase the levels of phosphorylated AKT and β-catenin to promote the invasion and migration of GC. These data collectively suggested that Hsp90ab1 inactivated AKT and Wnt/β-catenin signaling pathways.

**Fig. 9:**
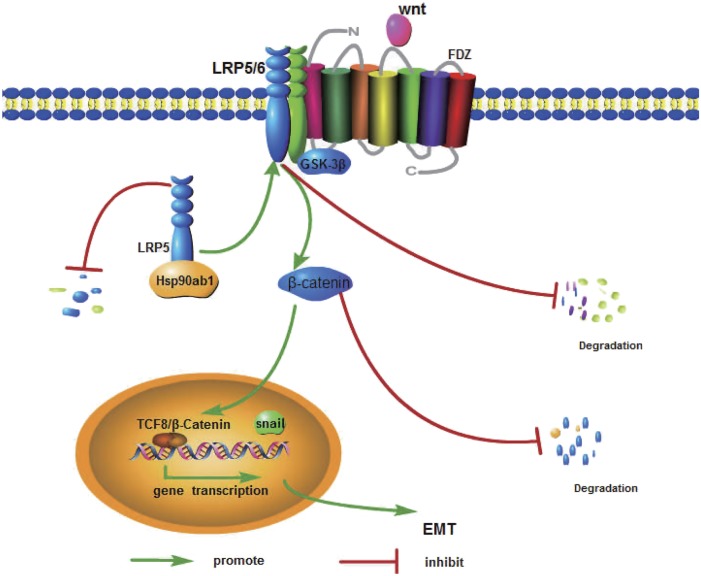
Schematic illustration of the molecular mechanism of Hsp90ab1 promoting the development of gastric cancer by a hypothetical model

In conclusion, this study investigated the potential role of Hsp90ab1 in GC cell invasion and metastasis. Our data suggested that Hsp90ab1 could bind to LRP5 and inhibit the ubiquitin degradation of LRP5 and subsequently activated the AKT and Wnt/β-catenin signaling pathways (Fig. 9). Hsp90ab1 may contribute significantly to develop specific drugs targets for GC patients.

## Materials and methods

### Tumor tissue sample

Two independent cohorts involving 332 people were involved in the study. In cohort 1, 150 primary GC patients who underwent radical surgery at Nanfang Hospital between 2013 and 2016 were enrolled in the study. All patients enrolled provided written informed consent prior to obtaining the study specimens of patients in accordance with the ethical protocols of the Ethics Committee of Nanfang Hospital, Southern Medical University. No patients received preoperative chemotherapy or radiotherapy before surgery. Among these tissue biopsies, 102 cases of the fresh-frozen tumor samples and matched adjacent non-tumor tissues were used for quantitative real-time (qRT)-PCR and 12 cases for western blotting analyses. Other 36 cases were used to investigate the correlativity between Hsp90ab1 and the biomarkers of EMT. For cohort 2, 182 cases of samples were enrolled for immunochemical stain for Hsp90ab1 using a gastric adenocarcinoma tissue microarrays (HStm-Ade180Sur-06 and HStmAde167Sur01; Shanghai Outdo Biotech), which was obtained from the National Engineering Center For Biochip. The clinicopathologic features of patients, including the age, gender, tumor location, size of primary tumor, tumor differentiation and tumor node metastasis classification, were recorded and archived in the National Engineering Center For Biochip.

### Cell line preparation

Human GC cell lines, including AGS, MKN45, MKN28, MGC803, NCI-N87 and SGC7901, were obtained from the Committee of Type Culture Collection of Chinese Academy of Sciences (Shanghai, China). Additionally, three human GC cell lines (BGC823, BGC803, MGC823), the gastric mucosal cell GSE-1 and HEK293 were kindly provided by the Department of Pathology of Nanfang Hospital, Southern Medical University.

### RNA extraction and qRT-PCR

RNA extraction and qRT-PCR were performed as previously described [56], with the following modifications. The primers were listed in Table S1. GAPDH was chosen as an internal quantitative reference. Each experiment was conducted in triplicate.

### Western blot analysis

The protein expression of cell lines as well as the fresh-frozen tumor samples and matched adjacent non-tumor tissue was performed in radio immunoprecipitation assay buffer (KeyGEN, Nanjing, China). 50 μg of protein lysate was loaded on a 10% SDS-PAGE gel and separated by electrophoresis. The proteins were transferred to polyvinylidene fluoride membranes (Millipore, Billerica, MA, USA). The membranes were incubated with various antibodies (Supplementary Table S2) overnight at 4 °C. The primary antibodies were detected using corresponding secondary antibodies (Supplementary Table S2). All experiments were repeated in triplicate, and the representative results were shown.

### Immunohistochemistry analysis

Immunohistochemistry (IHC) was performed as previously described [57], with the following modifications. The slides were incubated overnight with primary antibodies against Hsp90ab1 (1:500, ab203085, Abcam), E-cadherin (1:100, ZM-0092, Maixin Biotech), β-catenin (1:100, #8480, CST) and LRP5 (1:100, D260566, Sangon Biotech), Ki67 (1:100, ZM-0502, Maixin Biotech) at 4 °C. The images were analyzed as described in Supplementary Methods.

### Construction of plasmid and transfection

Expression of human Hsp90ab1 was knocked down using a lentivirus vector carrying a specific Hsp90ab1 short-hairpin RNA (shRNA) (Vigenebio, Maryland, USA) or siRNA (GenePharma, Shanghai, China), and LRP5 was knocked down using siRNA (GenePharma, Shanghai, China) as previously described [57, 58]. The target sequences were listed in Table S1 for details. Expression of human Hsp90ab1 was up-regulated using a lentivirus vector (Vigenebio, Maryland, USA) and LRP5 was up-regulated using a plasmid (Sino Biological Inc., Beijing, China) as previously described [57, 58]. Flag-LRP5 lysines (K) 143, K592, K806, K1231 and K1561 separately mutated to arginine (R), and plasmids termed “LRP5 K143R”, “LRP5 K592R”, “LRP5 K802R”, “LRP5 K1231R” and “LRP5 K1561R” mutants were generated by Mut Express II Fast Mutagenesis Kit V2 (Vazyme Biotech Co.,Ltd, Nanjing, China) and confirmed by sequencing. Plasmids and siRNAs were transient transfected into cells using Lipofectamine 2000, according to the manufacturer’s instructions (Thermo Fisher Scientific, USA).

### Cell proliferation, cell cycle, colony formation, wound healing, matrigel invasion assays

The Cell counting kit-8 (CCK-8), cell cycle, colony formation, cell migration and invasion assays were performed as described previously [59, 60].

### EdU incorporation assay

GC cell proliferation was determined in vitro using the Cell-Light ™ EdU staining kit (RiboBio, Guangzhou, China) according to manufacturer instructions. The proportion of positive cells in each well were counted.

### Oxaliplatin-sensitivity assay

The oxaliplatin-sensitivity of cells was measured as described previously [61].

### Xenograft tumor model

All animal experiments procedures were approved and conducted by the Southern Medical University Animal Care and Use Committee and the experiments were in accordance with the guidelines for the ethical treatment of animals. To determine the function of the Hsp90ab1 in tumor formation in vivo, cells stably expressing Hsp90ab1 were injected subcutaneously into nude mice. Tumor size was measured and calculated as previously described [57]. To explore the effects of Hsp90ab1 on tumor metastasis in vivo, cells stably expressing the indicated Hsp90ab1 or constructs were injected through the lateral tail vein of nude mice. 1 month later, the mice were sacrificed by cervical vertebra dislocation. Liver, lung and peritoneum were harvested, and prepared for hematoxylin and eosin and IHC staining as described above.

### Co-immunoprecipitation

Co-immunoprecipitation of proteins was performed as previously described [62], with the following modifications. Supernatants were incubated with protein A/G beads (Selleck Chemicals, Houston, USA) overnight at 4 °C to pre-clear the lysates. The pre-cleared lysates were incubated at 4 °C with primary antibody and protein A/G plus overnight according to the manufacturer’s instructions. The protein A/G-antibody-antigen complex was concentrated by centrifugation at 1000×*g* for 10 min at 4 °C and washed with PBS; the procedure was repeated three times. Immunoprecipitated proteins were then separated by SDS–PAGE, and visualized by Western blot and silver staining (Byeotime, Shanghai, China). The gels were digested for LC-MS/MS analysis as previously described [63], The detailed process was listed in the supplementary method.

### Immunofluorescence analysis

Cells were cultured on glass coverslips for 12 h and fixed with ethanol for 30 min at −20 °C. After fixation, cells were permeabilized with 0.25% Triton X-100 for 10 min at room temperature and blocked in 10% normal blocking serum at room temperature for 10 min, then incubated overnight at 4 °C with primary antibodies against Hsp90ab1 (1:200, ab203085, Abcam) and LRP5 (1:100, sc390267, Santa Cruz), and β-catenin (1:200, #8480, CST). The next day, slides were incubated with Alexa Fluor 488 and Alexa Fluor 594 labeled secondary antibodies (1:1000, Proteintech Group Inc, Wuhan, China) for 1 h at room temperature. To visualize nuclei, slides were incubated with 6-diamidino-2-phenylindole (DAPI; KeyGEN, Nanjing, China).

### Glutathione S-transferase protein pull-down assay

GST-pET-41a( + )-Hsp90ab1 (1–231a), (232–263a), (264–609a), (610–621a), (622–724a), (1–621a), (232–724a), and (1–263/610–724a), GST-pET-41a(+)-LRP5 (1–642a), (1–1255a), (1256–1408a) and (1409–1615a) (Vigenebio, Maryland, USA) were transformed into Escherichia coli strain BL21 (DE3) and induced for the expression of GST or GST-fusion protein by IPTG. And these proteins were purified using the Beaver beads TM GSH kit (Beaver Biosciences Inc, Suzhou China) according to the manufacturer’s instructions. Pull-down assays were performed by incubating GST fusion protein with the cell lysates of HEK293T cells which were transfected with Flag-LRP5 at 4 °C for 4 h. Then the bead-bound protein complexes were then washed and detected by western blot.

### Protein ubiquitination assay

Hsp90ab1 expression lentiviral vector or empty lentiviral vector were transfected into BGC823 cells. Cells were incubated in the presence or absence of 30 μM MG132 (Selleck, Houston, USA) for 24 h and lysed in RIPA buffer supplemented with proteinase inhibitor. Then immunoprecipitation was carried out with anti- Hsp90ab1 or anti-IgG antibodies following the protocol above. The immunoprecipitated proteins were subjected to western blot using anti-ubiquitin (Proteintech Group Inc., Wuhan, China) to evaluate the ubiquitination level.

### Statistical analysis

All statistical analyses were performed using SPSS version 19.0 software (SPSS, Chicago, IL, USA). The correlation between the protein expression and clinicopathological factors in GC tumor tissue and the paired normal tissue was determined by Pearson’s chi-square test. Survival curves were plotted according to the Kaplan–Meier method and were compared with the log-rank test. The volumes of xenograft tumor in nude mice from the experimental and control groups were compared with Student’s t-test. The statistical analysis of cell proliferation curve, and migration rate between different cell groups was carried out by One-way ANOVA and the *χ*^2^ test. Values of *P* *<* 0.05 were considered statistically significant.

## Electronic supplementary material

Supplementary method
