# Supplementary material for: Hsp90ab1 stabilizes LRP5 to promote epithelial–mesenchymal transition via activating of AKT and Wnt/β-catenin signaling pathways in gastric cancer progression
Source: Oncogene. 2018 Oct 10;38(9):1489–507. doi: 10.1038/s41388-018-0532-5 (PMC6372478; doi:10.1038/s41388-018-0532-5)
Supplement: Supplementary file 1 — Supplementary method [file 41388_2018_532_MOESM1_ESM.docx]

**Evaluation of immunohistochemical staining for Hsp90ab1.**

The total Hsp90ab1 immunostaining score was calculated by summing up the percentage positivity of stained tumour cells and the staining intensity. The percent positivity was scored on a scale of 0-4 as follows: ‘‘0,’’ 0%; ‘‘1,’’ 1–25%; ‘‘2,’’ 26–50%; ‘‘3,’’ 51–75% and ‘‘4,’’ >75%. The staining intensity was scored from 0 to 3, with 0 for no staining, 1 for weakly stained, 2 for moderately stained, and 3 for strongly stained. Both of the percent positivity of cells and the staining intensity were scored by two independent pathologists who were blinded to the patients' clinicopathological data. Then the final staining score for Hsp90ab1 was calculated with the sum of the staining-intensity and staining-extent scores, which ranged from 0 to 7. Tumors with a final staining score 0-3 were defned as those with low expression of Hsp90ab1, and tumors with a final staining score of 4-7 were defned as those with high expression of Hsp90ab1.

**The protocol for LC-MS/MS analysis.**

In gel digestion:

The samples were washed twice with 200 μL of Nanopure water and then decolored with 200 μL of de-stained solution at room temperature for 30 min. The pieces were dehydrated with 200 μL of dehydration solution 1 for 30 min, and then with 200 μL of dehydration solution 2 for 30 min. Then dehydration solution 2 was discarded. Traces of remaining dehydration solution 2 were removed by vacuum drying in SpeedVac for 5 min. The gel pieces were rehydrated in 100 μL of reduction solution 1 and incubated at 57 °C for 1 h. The solution was discarded and the pieces were incubated in 100 μL of freshly prepared reduction solution 2 at room temperature in the dark for 20 min. The pieces were washed in 400 μL of imbibition solution for 10 min and, then, were dehydrated, first in 200 μL of dehydration solution 1 for 30 min with intermittent mixing and then with 200 μL of dehydration solution 2 for 30 min. Dehydration solution 2 was discarded and the gels were rehydrated in 10 µL digest solution for 30 min, then, 20 µL cover solution was added for digestion 16 hours at 37 ºC. The supernatants were transferred into another tube and the gels were extracted once with 50 µL extraction buffer at 37℃ for 30 min. The peptide extracts and the supernatant of the gel spot were combined and then completely dried.

LC-MS/MS Analysis:

The samples were re-suspended with Nano-RPLC buffer A and the online Nano-RPLC was employed on the Eksigent nanoLC-Ultra™ 2D System (AB SCIEX). The samples were injected into a C18 nanoLC trap column(100µm×3cm, C18, 3µm, 150Å) and washed by Nano-RPLC Buffer A(0.1%FA, 2%ACN) at 2μL/min for 10 mins. And an elution gradient of 5-35% acetonitrile (0.1%formic acid) in 90 mins gradient was used on an analytical ChromXP C18 column (75μm x 15 cm, C18, 3μm 120 Å) with spray tip.

Data acquisition was performed with a Triple TOF 5600 System (AB SCIEX, USA) fitted with a Nanospray III source (AB SCIEX, USA) and a pulled quartz tip as the emitter (New Objectives, USA). Data were acquired using an ion spray voltage of 2.5 kV, curtain gas of 30 PSI, nebulizer gas of 5 PSI, and an interface heater temperature of 150℃. For information dependant acquistion (IDA), survey scans were acquired in 250 ms and as many as 35 product ion scans were collected if they exceeded a threshold of 150 counts per second (counts/s) with a 2+ to 5+ charge-state. The total cycle time was fixed to 2.5 s. A rolling collision energy setting was applied to all precursor ions for collision-induced dissociation (CID). Dynamic exclusion was set for ½ of peak width (18 s). And the precursor was then refreshed off the exclusion list. Based on combined MS and MS/MS spectra, proteins were successfully identified based on 95% or higher confidence interval of their scores in the MASCOT V2.3 search engine (Matrix Science Ltd., London, U.K.), using the following search parameters: cow-lacbobacillus casel mix database; trypsin as the digestion enzyme; two missed cleavage site; fixed modifications of Carbamidomethyl (C); partial modifications of Acetyl (Protein N-term), Deamidated (NQ), Dioxidation (W), Oxidation (M) Phospho (ST) and Phospho (Y); ±15 ppm for precursor ion tolerance and ±0.15 Da for fragment ion tolerance.
